# Surgical approaches for brainstem tumors in pediatric patients

**DOI:** 10.1007/s00381-015-2799-y

**Published:** 2015-09-09

**Authors:** Sergio Cavalheiro, Kaan Yagmurlu, Marcos Devanir Silva da Costa, Jardel Mendonça Nicácio, Thiago Pereira Rodrigues, Feres Chaddad-Neto, Albert L. Rhoton

**Affiliations:** Department of Neurosurgery, Pediatric Oncology Institute, Federal University of Sao Paulo, Sao Paulo, Brazil; Department of Neurosurgery, University of Florida, College of Medicine, Gainesville, FL USA; Department of Neurosurgery, Vascular Division, Federal University of Sao Paulo, Sao Paulo, Brazil; Department of Neurosurgery, Federal University of Sao Paulo, Sao Paulo, Brazil; Rua Botucatu, 591, conj 41, CEP: 04023-062 Sao Paulo, SP Brazil

**Keywords:** Pediatric brainstem gliomas, Brainstem surgery, Safe entry zone, White fiber anatomy, Supratrigeminal approach, Low-grade astrocytoma

## Abstract

**Purpose:**

To analyze the pathways to brainstem tumors in childhood, as well as safe entry zones.

**Method:**

We conducted a retrospective study of 207 patients less than 18 years old who underwent brainstem tumor resection by the first author (Cavalheiro, S.) at the Neurosurgical Service and Pediatric Oncology Institute of the São Paulo Federal University from 1991 to 2011.

**Results:**

Brainstem tumors corresponded to 9.1 % of all pediatric tumors operated in that same period. Eleven previously described “safe entry zones” were used. We describe a new safe zone located in the superior ventral pons, which we named supratrigeminal approach. The operative mortality seen in the first 2 months after surgery was 1.9 % (four patients), and the morbidity rate was 21.2 %.

**Conclusions:**

Anatomic knowledge of intrinsic and extrinsic brainstem structures, in association with a refined neurosurgical technique assisted by intraoperative monitoring, and surgical planning based on magnetic resonance imaging (MRI) and tractography have allowed for wide resection of brainstem lesions with low mortality and acceptable morbidity rates.

## Introduction

The brainstem is one of the most complex structures in the human body and contains the most complex intracranial anatomy [55]. This compact, midline organ is protected anteriorly by the clivus, laterally by the petrous part of temporal bone, superiorly by the diencephalon, and posteriorly by the cerebellum. All motor, sensory, sympathetic, and parasympathetic brain functions are integrated and travel through the brainstem. The structural complexity of the brainstem makes surgical procedures there in extremely difficult and require a perfect technique. Brainstem tumors are more common in children and represent up to 18 % of childhood brain tumors and 25 % of posterior cranial fossa tumors. There is no gender predilection. The mean age incidence for these tumors occurs around age 5 to 10 years [54]. A second peak of incidence is seen in adults between 30 to 40 years of age. In recent years, several articles have been published on brainstem anatomy and “safe entry zones.” Most of these are related to cavernoma surgery and few are related to brainstem tumor surgical approaches in children [9, 19, 22, 26, 53]. The aim of this study was to review the described surgical approaches and those conducted by the first author (Cavalheiro, S.) of this article on the basis of 207 patients aged less than 18 years who underwent brainstem tumor surgery.

## Classification

The various arrangements of fibers, Virchow-Robin spaces, and structures comprising the brainstem occasionally allow tumors to grow considerably with few symptoms. This may permit diffuse pontine tumors to grow within the pons without infiltrating the mesencephalon or medulla. When midbrain tumors grow, they spread towards the thalamus and do not infiltrate the pons. Tumors of the medulla tend to grow into the fourth ventricle without invading the pons, or grow caudally towards the spinal cord.

Many classifications have been proposed for brainstem tumors. We used that proposed by Choux et al.: type I, diffuse brainstem gliomas; type II, focal intrinsic tumors (solid or cystic); type III, exophytic; and type IV, cervicomedullary [14, 15, 17].

### Diffuse tumors (type I)

Diffuse tumors are the most common, representing up to 80 % of brainstem tumors. They simultaneously affect multiple nuclei and pathways, and characteristically cause bilateral paralysis of cranial nerves VI and VII, progressing to hemiparesis and tetraparesis. They present with rapid clinical evolution, and as far as histopathology is concerned, most are malignant astrocytomas (WHO) grade III or IV. Radiologically, they are characterized by pontine enlargement with an entrapped basilar artery. They are hypointense on T1 magnetic resonance imaging (MRI), hyperintense on T2, hyperintense on FLAIR, and exhibit minimal contrast enhancement [6, 44].

Survival of these patients is short, and most die within the first 2 years after diagnosis. However, a few cases may respond to chemotherapy and radiation. There is no difference in prognosis using conventional or multi-fractionated radiotherapy. Metastases of the neuraxis may occur in 5 to 30 % of cases [10, 18, 25].

Stereotactic biopsy of diffuse brainstem tumors has been performed in a few centers. Its use is important mainly for molecular biology studies [52, 57, 62]. However, biopsies are associated with some complications. Pincus et al. (2006) [49] conducted a retrospective study of 182 stereotactic biopsy cases from 13 published reports of diffuse pontine lesions in children. They noted that tumor diagnosis was verified in 75 to 100 % of cases. In 87 % of cases, the lesions were gliomas, while the remaining 13 % were primitive neuroectodermal tumors, neurocytomas, ependymomas, and demyelinating lesions. Morbidity ranged from 0 to 16 %, and mortality reached 5 %. Therefore, biopsy is indicated only in cases with non-characteristic images or in molecular biology research centers [32, 59]. Stereotactic biopsies can be performed through entry points in the frontal region or through the posterior fossa.

### Focal tumors (type II)

Focal tumors behave differently from diffuse tumors. They are slow-growing lesions, and the symptomatology is indolent. They may be solid or cystic and, contrary to diffuse tumors, local lesions have well-delimited borders. Less edema is associated with focal tumors, which are mainly low-grade gliomas. They are usually hypointense on T1, with diffuse tumoral enhancement. Impregnation with gadolinium varies in focal tumors; however, homogeneous enhancement is highly suggestive of pilocytic astrocytoma [29].

If the tumor is superficial, surgery is advised; however, if it is deep, the treatment should be conservative, in the expectation that the tumor itself may provide an “opening door” for its resection. The use of tractography has allowed for better choices regarding surgical approaches to these tumors. Tumors in the quadrigeminal plate are usually focal and mostly pilocytic astrocytomas [33].

### Exophytic tumors (type III)

Exophytic tumors are more accessible surgically. They tend to be large tumors with a large component out of the brainstem, facilitating surgery. They may have a cystic component, another factor that facilitates its resection. They are mostly low-grade astrocytomas [48].

### Cervicomedullary junction tumors (type IV)

Cervicomedullary junction tumors often present as an exophytic growth, allowing surgeons direct access without incising the brainstem. These lesions usually do not infiltrate the pons and grow cranially into the fourth ventricle. They may extend caudally into the spinal cord. When growing toward the fourth ventricle, hydrocephalus may occur early on. When growing towards the spinal cord, they may produce syringomyelia, due to changes in cerebrospinal fluid dynamics. Although surgical approaches are facilitated by topography, these are cases that most often progress with serious morbidity. Postoperatively, these patients may have difficulty breathing, resulting in long periods of assisted mechanical ventilation, and swallowing difficulties, which in turn may cause severe aspiration pneumonia. Patients may require tracheostomy and gastrostomy, necessitating speech therapy. The use of electrophysiological monitoring during surgery has helped to prevent those complications [16].

## Patients and methods

From 1991 to 2011, 303 patients younger than 18 years with brainstem tumors were treated by the Neurosurgical Service and Pediatric Oncology Institute of the Federal University of Sao Paulo. Of these, the first author of this article surgically treated 207. The remaining 96 cases were diffuse tumors. Here, we describe the surgical approaches and relevant extrinsic/intrinsic anatomical points used.

**Fig. 1:**
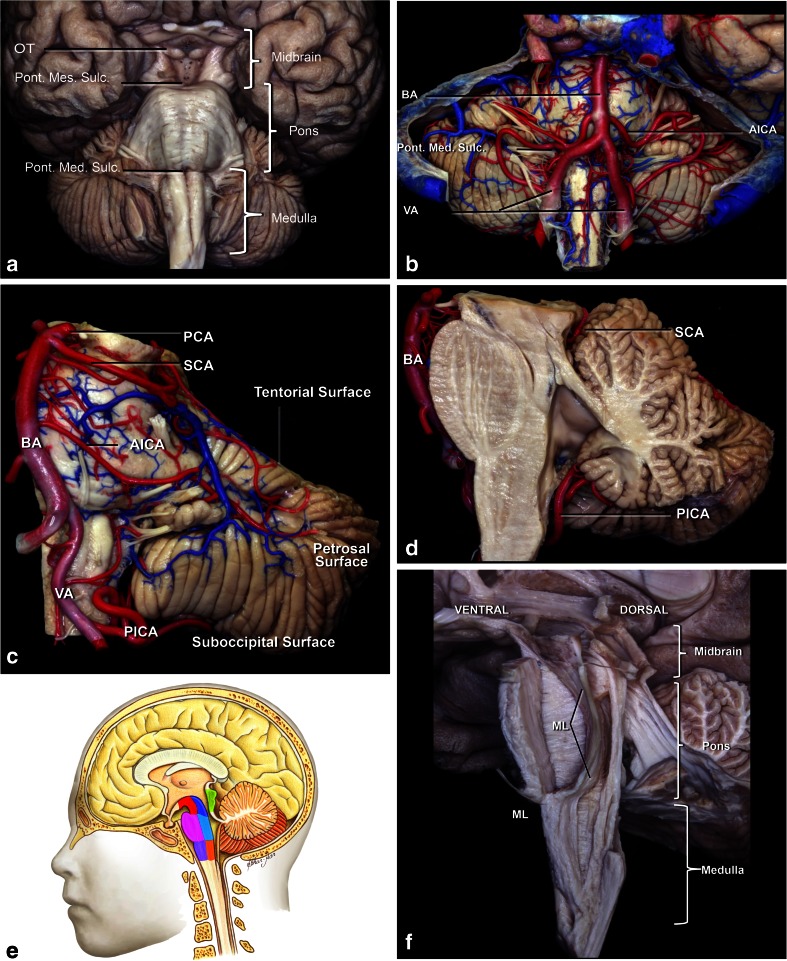
**a**. The brainstem surface anatomy. The brainstem is divided into three parts: the midbrain, pons, and medulla. The midbrain is limited superiorly by the sulcus between optic tract (OT) and crus cerebri, and inferiorly by the pontomesencephalic sulcus (Pon. Mes. Sulc.). The pons is positioned between the pontomesencephalic sulcus above and pontomedullary sulcus (Pont. Med. Sulc.) below. The medulla extends from the pontomedullary sulcus to the exit zone of the C1 nerve root. **b**, **c** ***,*** **d** The brainstem vascularization. The vertebral arteries (VA) meet to form the basilar artery at the level of the pontomedullary sulcus. The basilar artery (BA) gives rise to posterior cerebral arteries (PCA) at the level of the pontomesencephalic sulcus. The cerebellum has three surfaces: tentorial, petrosal, and suboccipital. The suboccipital surface of the cerebellum and medulla is supplied by the posteroinferior cerebellar artery (PICA). The petrosal surface of the cerebellum and pons are supplied by AICA and basilar artery perforators, while the midbrain and tentorial surface of the cerebellum is supplied by the superior cerebellar artery (SCA) branches. **e** Another division of the brainstem into seven compartments: the ventral, central, and dorsal midbrain; ventral and dorsal pons; and ventral and dorsal medulla. **f** Anatomical division of the brainstem according to course of the medial lemniscus (ML). In front of the ML can be considered the ventral brainstem, and behind the medial lemniscus as the dorsal brainstem

The brainstem is divided into three segments: the midbrain, pons, and medulla. In order to choose the most effective and safe approaches for removal of the lesions, we divided the brainstem into seven portions (Fig. 1e).

The midbrain was divided into three parts: anterior, central, and posterior. The anterior segment is delimited posteriorly by the substantia nigra. The central midbrain extends from the substantia nigra to the aqueduct. The posterior part is restricted to the quadrigeminal plate. The pons was divided into two segments: anterior and posterior, or ventral and dorsal. Similarly, the medulla was divided into anterior and posterior, or ventral and dorsal, partitions. Detailed knowledge of the brainstem extrinsic and intrinsic anatomy is essential to avoid morbidity during the surgical approaches.

### Midbrain

**Fig. 2:**
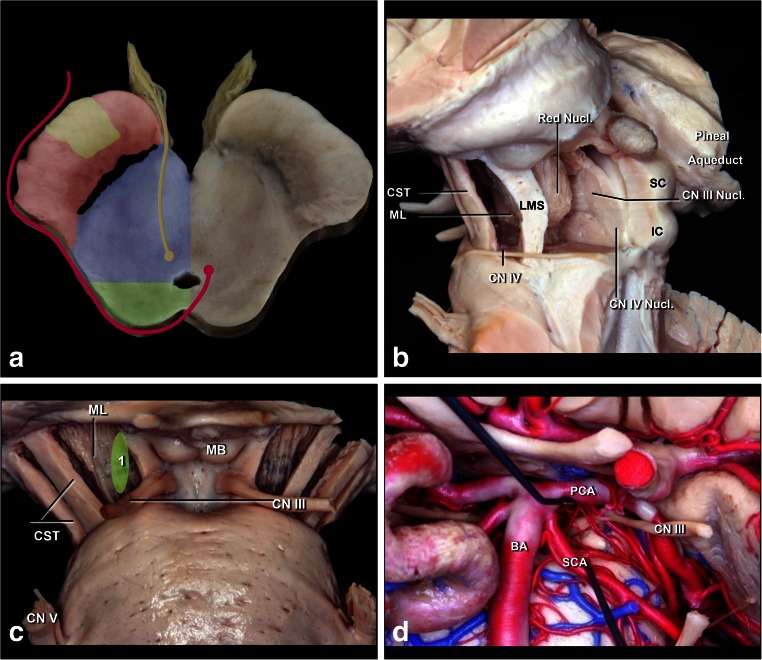
**a** Schematic representation of the midbrain at the level of the oculomotor nerve. The substantia nigra and medial lemniscus is the border between ventral (*anterior*) and central parts of the midbrain, while the level of the cerebral aqueduct is the border between central (*blue*) and dorsal (*posterior*) midbrain (*green*). The corticospinal tract (*yellow*) in the ventral midbrain (*red*); nuclei and courses of the CN III (*yellow*) and IV (*red*) in the central (*blue*) and dorsal midbrain. **b** Lateral view of the midbrain. The ventral (anterior) midbrain, which contains the corticospinal tract (CST), is situated in front of the medial lemniscus (ML) and substantia nigra. The central midbrain containing the red, oculomotor and trochlear nuclei is positioned between medial lemniscus and aqueduct. The dorsal (*posterior*) midbrain composed of superior (SC) and inferior colliculi (IC) is positioned behind the cerebral aqueduct. The nuclei of the CN III and IV are located just ventral to the aqueduct. **c** Anterior view of midbrain. *1* Perioculomotor entry zone is bordered medially by exit point of the CN III and laterally by the corticospinal tract (CST). **d** The perioculomotor zone is limited by the posterior cerebral artery (PCA) superiorly and by the superior cerebellar artery (SCA) inferiorly

The midbrain is separated from the diencephalon by a sulcus between the optic tract and cerebral peduncle, and from the pons by the pontomesencephalic sulcus. Anatomically, three structures should be widely recognized in the midbrain: the pyramidal tract located in the anterolateral portion of the midbrain, nuclei of the third and fourth cranial nerves [63] (Figs. 1 and 2a). The level of the nucleus of the third cranial nerve is at the lower half of the superior colliculus and the upper half of the inferior colliculus. The trochlear nucleus lies caudal to the inferior half of the inferior colliculus. These nuclei are positioned adjacent to the midline and on average 9.5 mm medial to the surface of the lateral mesencephalic sulcus. The mesencephalic lateral sulcus runs from the medial geniculate body superiorly to the pontomesencephalic sulcus inferiorly. This sulcus is considered the posterior limit of the ventral lateral midbrain. The third cranial nerve has a long course along all the central portion of the mesencephalon while the fourth cranial nerve has a smaller intrinsic portion and runs through the contralateral cerebellomesencephalic fissure. The third cranial nerve exits at the medial sulcus of the midbrain peduncle and goes toward the oculomotor triangle, towards its entry into the cavernous sinus. The midbrain receives its blood supply through mesencephalic perforating basilar artery branches. They are divided into anteromedial, anterolateral, lateral, and posterior branches. The anteromedial branches are divided into lateral and medial. The medial branches supply the red nucleus, periaqueductal gray matter, and the third and fourth cranial nerve nuclei. The lateral branches supply the medial lemniscus, substantia nigra, and superior cerebellar peduncle. The anterolateral midbrain arteries are called peduncular branches, and supply the crus, substantia nigra, and medial lemniscus. They arise from many arteries, including the collicular, medial posterior choroidal, posterior communicating artery, superior cerebellar artery, and anterior choroidal artery. Posterior arteries are formed by branches of superior cerebellar artery and by collicular arteries, and they form a plexus involving the quadrigeminal plate [55].

### Anterior midbrain

**Fig. 3:**
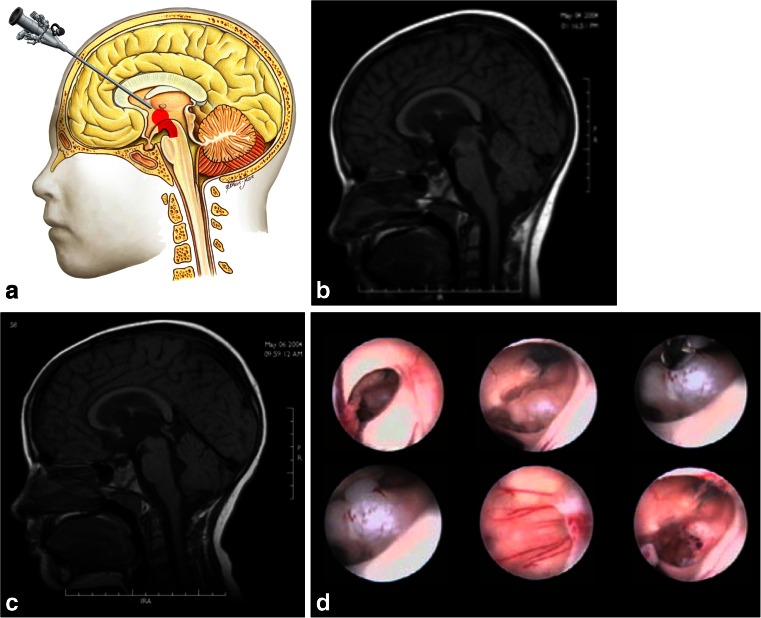
**a** Endoscopic access to tumors located in the anterior and superior portion of the midbrain. **b** An 8-year-old patient with intracranial hypertension. A lesion is present in the anterior and superior topography of the midbrain. **c** 3-year follow-up after complete removal of the lesion by endoscopy, with patency of the cerebral aqueduct. Diagnosis was low-grade astrocytoma. **d**. Intraoperative endoscopic view of the exophytic tumor in the midbrain and after complete resection. After passing through the foramen of Monro, the lesion was seen and removed at the floor of the third ventricle

**Fig. 4:**
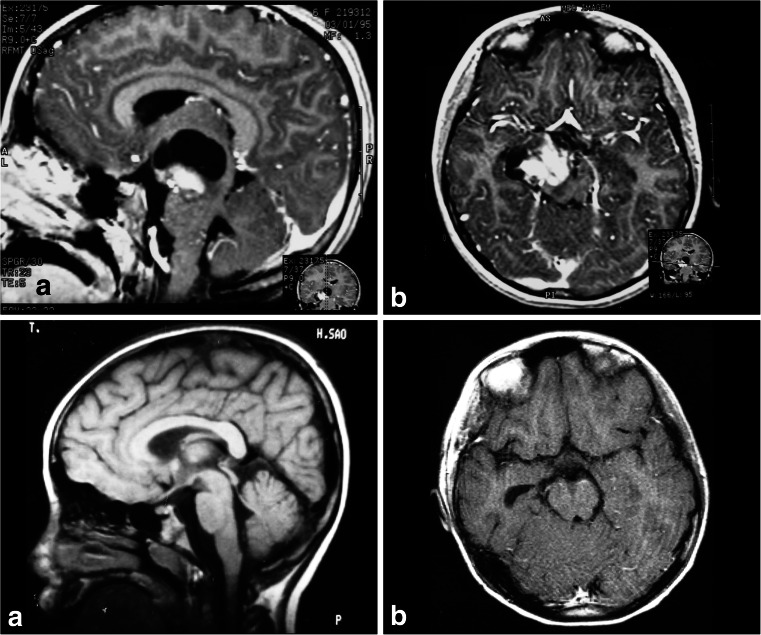
**a**, **b** 4-year-old girl admitted with a right Weber Syndrome with a solid/cystic anterior mesencephalic lesion with expansion towards the interpeduncular cistern. **c**, **d** 6-year follow-up showing total resection by fronto-orbito zygomatic approach (pilocytic astrocytoma)

Tumors in the anterior portion of the midbrain usually grow in two directions: toward the third ventricle and toward the interpeduncular cistern. For tumors growing into the third ventricle, an interfornicial, transcallosal, transchoroidal, or transforaminal approach is used. When the lesion is less than 2 cm in size, a neuroendoscope coupled to an ultrasonic aspirator can be used for removal of the tumor, most of which are low-grade astrocytomas (Fig. 3). When these tumors grow toward the interpeduncular cistern, they usually present with Weber syndrome (third cranial nerve impairment and contralateral hemiparesis) (Fig. 4).

**Fig. 5:**
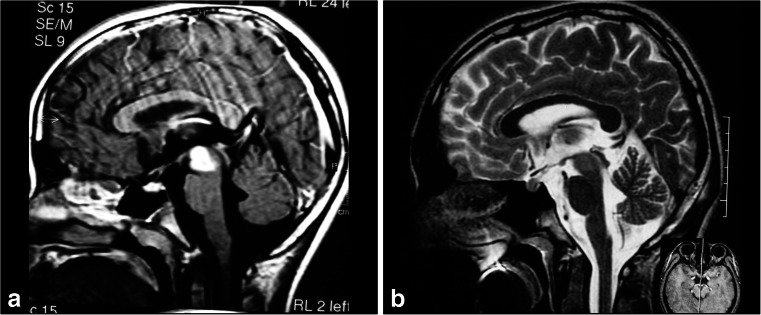
**a**. A 7-year-old patient with diplopia and paresis of the oculomotor nerve on the right side. MRI shows a focal and intrinsic lesion parallel to the intrinsic course of the third nerve in the midbrain. **b**. Follow-up 5 years after gross total resection showing no residual lesion

Anterior and anterolateral lesions of the midbrain can be approached by the transsylvian pathway with a classic pterional, orbito-fronto-zygomatic, or temporal route. Through those routes, it is possible to combine approaches via a temporopolar approach (pre-temporal or transtentorial subtemporal). The temporopolar approach was described by Sano in 1980 [58]. It allows for an opening over the temporal lobe in the posterior-superior direction and visualization of the anterolateral interpeduncular fossa. Another approach to be used is the subtemporal transtentorial, as described by Krause in 1911 [36]. This approach increases the risk of venous infarct due to the injury to the vein of Labbe complex, and ophthalmoparesis due to third and fourth cranial nerve injury along the tentorial incisure. On the other hand, this approach allows an excellent view of the incisural space. The incision ensures good exposure of the basilar artery, interpeduncular cistern, brainstem, and rostral ventral surface of the pons. A “fairly safe” entry zone into the anterolateral midbrain, described by Bricolo and Turazzi [7], has been proposed since the fibers of the corticospinal tract occupy only the intermediate three-fifths of the peduncle (Fig. 2c). This narrow window is delimited above by the posterior cerebral artery, below by the superior cerebellar artery, medially by the emergence of cranial nerve III and the basilar artery, and laterally by the pyramidal tract (Fig. 5). Tumors here are often exophytic, making it unnecessary to enter the brainstem, and the tumor can be incised at the exit point of the lesion. In cases of focal tumors, where it is necessary to incise the brainstem, a diamond-tipped scalpel is used. Bipolar coagulation is not used. Incisions parallel and lateral to the third cranial nerve are made to prevent pyramidal tract injury. Access in this way is termed perioculomotor. Great care must be taken with this strategy to avoid injury of the red nucleus, pyramidal tract, and the oculomotor nerve.

### Central midbrain

**Fig. 6:**
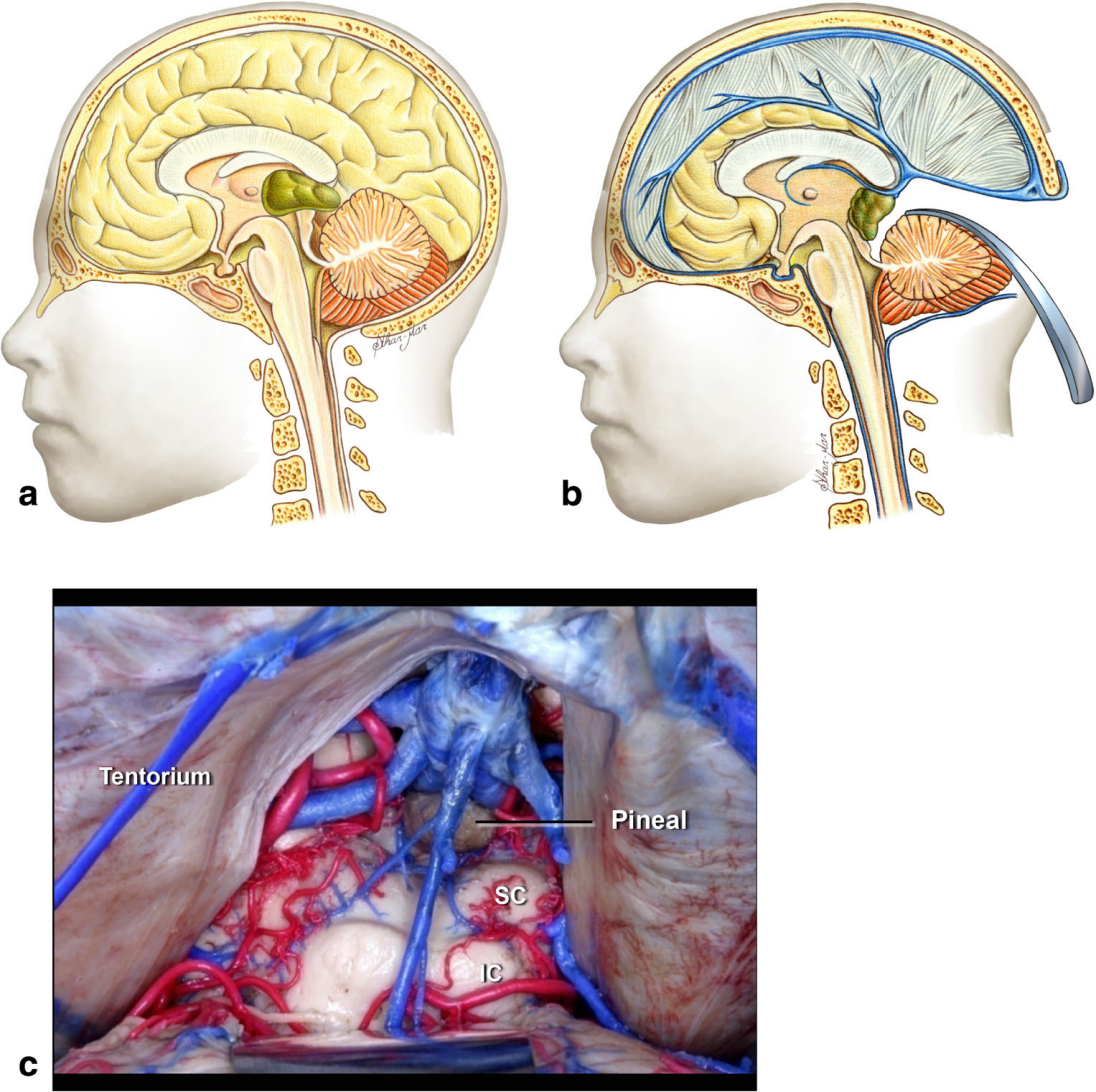
**a**. Tumors located at the central portion of the midbrain and growing towards the pineal region. **b** Infratentorial supracerebellar approach. **c** Anatomical view of pineal region via the infratentorial supracerebellar route. *SC* superior colliculus, *IC* inferior colliculus

**Fig. 7:**
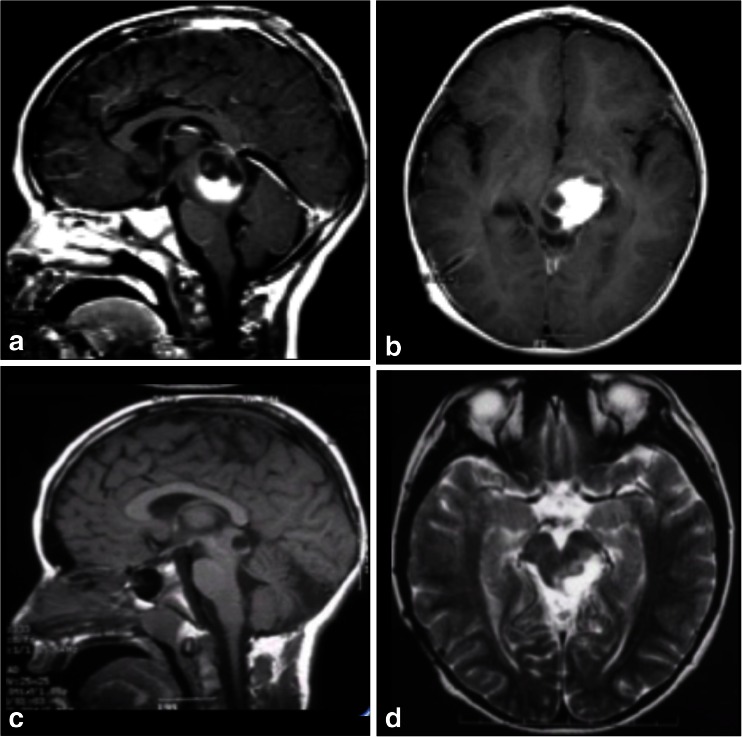
**a**, **b** A solid cystic tumor at the central portion of the midbrain with gadolinium enhancement growing towards the pineal region was removed by a central infratentorial supracerebellar approach. **c**, **d** 10-year follow-up shows no evidence of the lesion, which was a pilocytic astrocytoma

**Fig. 8:**
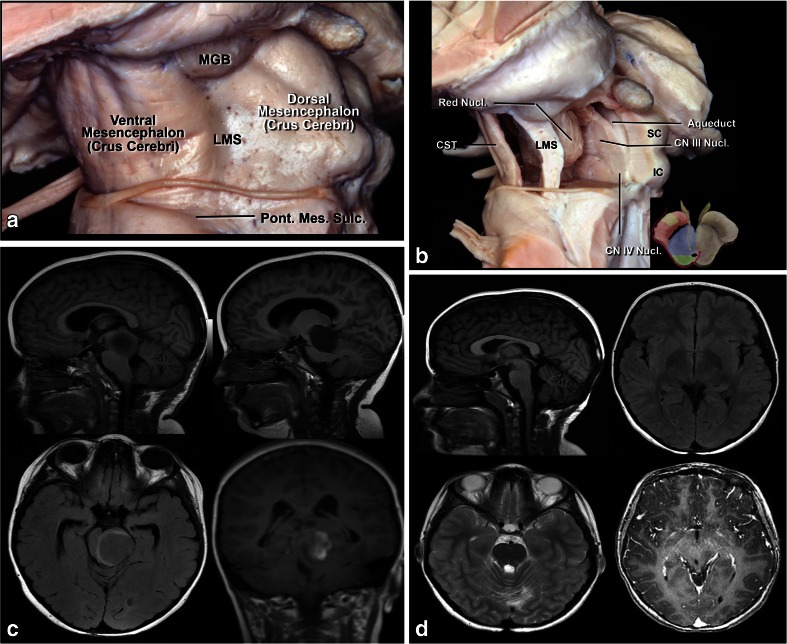
**a** Lateral view of the midbrain. The lateral mesencephalic sulcus (LMS) runs on the surface of the midbrain extending from the medial geniculate body (MGB) above to the pontomesencephalic sulcus (Pont. Mes. Sulc.) below. The LMS extends along the lateral edge of the medial lemniscus (ML). **b** The ML divides the midbrain into ventral (*anterior*) and dorsal (*posterior*) parts. Neurocritical structures at entry through the LMS are the corticospinal tract in the anterior midbrain, the red, oculomotor and trochlear nuclei in the central (tegmentum) midbrain, nuclei of the superior and inferior colliculus in the posterior (*dorsal*) midbrain. The oculomotor nucleus is positioned at the level of the inferior half of the superior colliculus and superior half of the inferior colliculus in the midline, and the trochlear nucleus is positioned at the level of the inferior half of the inferior colliculus in the midline. **c** Lateral lesion in the central midbrain approached via infratentorial supracerebellar route along the lateral sulcus of the mesencephalon. **d** Five-year follow-up after complete resection showing no evidence of the lesion, a pilocytic astrocytoma

Tumors located in the intermediate midbrain can also grow in two directions: toward the pineal region or into the fourth ventricle. When they grow toward the fourth ventricle, the suboccipital telovelar approach is used. When they grow towards the pineal region, we use the path suggested by Krause in 1911 [37] and popularized by Stein [60], which is the suboccipital infratentorial supracerebellar route (Figs. 6 and 7), further divided into median or paramedian. Patients are usually in the sitting position for this approach. An extensive posterior fossa craniotomy with removal of the C1 arch is performed. This approach allows for wide movement of the cerebellum. After coagulation of the vermian veins, the cerebellum drops to allow access to the midbrain. When lesions are medial, we also coagulate the precentral cerebellar vein. When lesions are lateral, there is no need to coagulate this vein (Fig. 8). Care should be taken in this region as the fourth cranial nerve is directly below the inferior colliculus. With exophytic lesions, there is no need to incise the brainstem, and we can directly approach the tumor, except for small cavernomas for which we used three access routes: upper and lower pericollicular, and through the lateral mesencephalic sulcus.

From the pericollicular access point, two “safe zones” can be accessed: an incision made in the midbrain below the inferior colliculus, or infracollicular access, and above the trochlear nerve, or supracollicular access. In the supracollicular approach, a transverse incision is made just above the superior colliculus and should be limited by the aqueduct. Further extension in this approach can damage the nuclei of cranial nerves III and IV, as well as the medial longitudinal fasciculus. With infracollicular access, a transverse incision between the trochlear nerve and the lower edge of the inferior colliculus is performed. As for the supracollicular route, an incision deeper than the cerebral aqueduct will damage the nuclei of the third and fourth cranial nerves and the medial longitudinal fasciculus. More lateral extensions of this incision will damage the superior cerebellar peduncle, the trigeminal mesencephalic tract, and the decussation of the superior cerebellar peduncle.

For lesions extending towards the fourth ventricle, we can incise the quadrangular lobe of the cerebellum for greater access to the cerebellar mesencephalic fissure. In this approach, it is essential to have a spatial imagination of the third and fourth nerves nuclei, as well as of their course inside the midbrain.

The ultrasonic aspirator is extremely important in that situation, and the color of the tumor is also most helpful in achieving total resections. Some tumors, however, are the same color as the brainstem, and then the surgeon has to rely on the position of the fibers, as well as on the tumor texture and circulation. Tumors are usually softer than the normal brain stem and also more vascularized, which makes it easier to remove them.

### Posterior (dorsal) midbrain

Posterior midbrain or quadrigeminal plate is the name given to the portion of the midbrain that is posterior to the cerebral aqueduct. The tumors of the quadrigeminal plate are the smallest brain tumors liable to kill the patient from hydrocephalus. They account for approximately 5 % of pediatric tumors of the brain stem [24]. They are usually indolent lesions and the treatment is limited to the treatment of hydrocephalus. Most of the time, these tumors are isointense on T1-weighted and hyperintense on T2-weighted images. Up to 19 % of cases may have gadolinium-enhanced MRI [23].

**Fig. 9:**
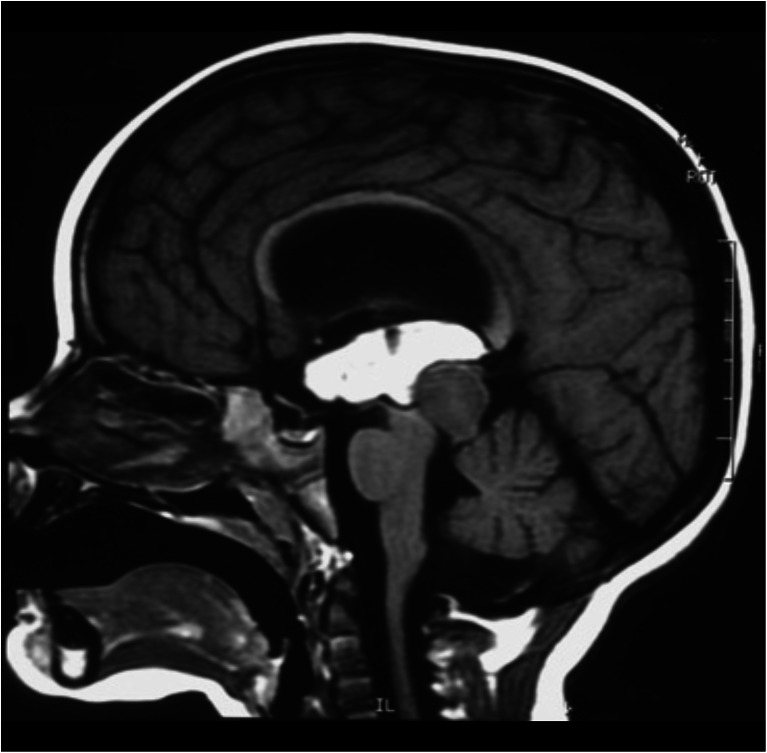
Intraventricular hemorrhage after endoscopic biopsy of a quadrigeminal plate lesion—pilocytic astrocytoma

The hydrocephalus is best treated by endoscopic third ventriculostomy [38]. Endoscopic biopsy should be avoided due to the possibility of a hemorrhage, distal from the biopsy area (Fig. 9), most of such lesions being low-grade astrocytomas such as pilocytic and non-pilocytic astrocytomas, mixed gliomas, and rarely more aggressive tumors as anaplastic astrocytomas [50].

**Fig. 10:**
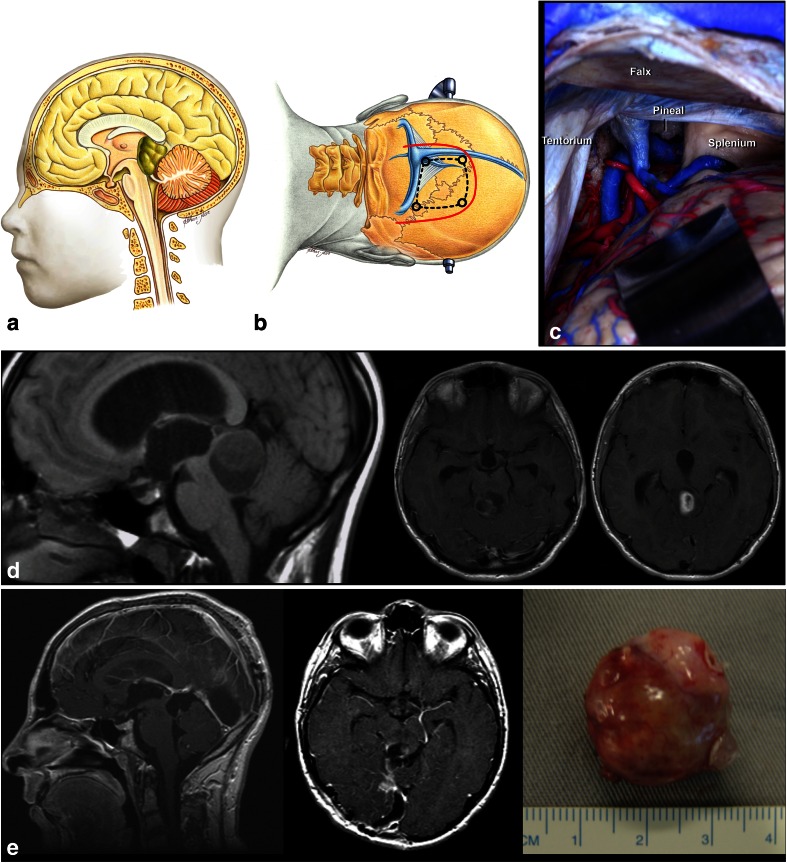
**a** Tumor in the quadrigeminal plate growing towards the fourth ventricle. **b** Transtentorial occipital approach. **c** Right occipital transtentorial view. The pineal gland and splenium are exposed. **d**. Quadrigeminal plate tumor growing towards the fourth ventricle, operated via transtentorial-occipital approach. **e**. Postoperative imaging 9 years after gross total resection of the lesion, showing no evidence of tumor, which was a pilocytic astrocytoma

**Fig. 11:**
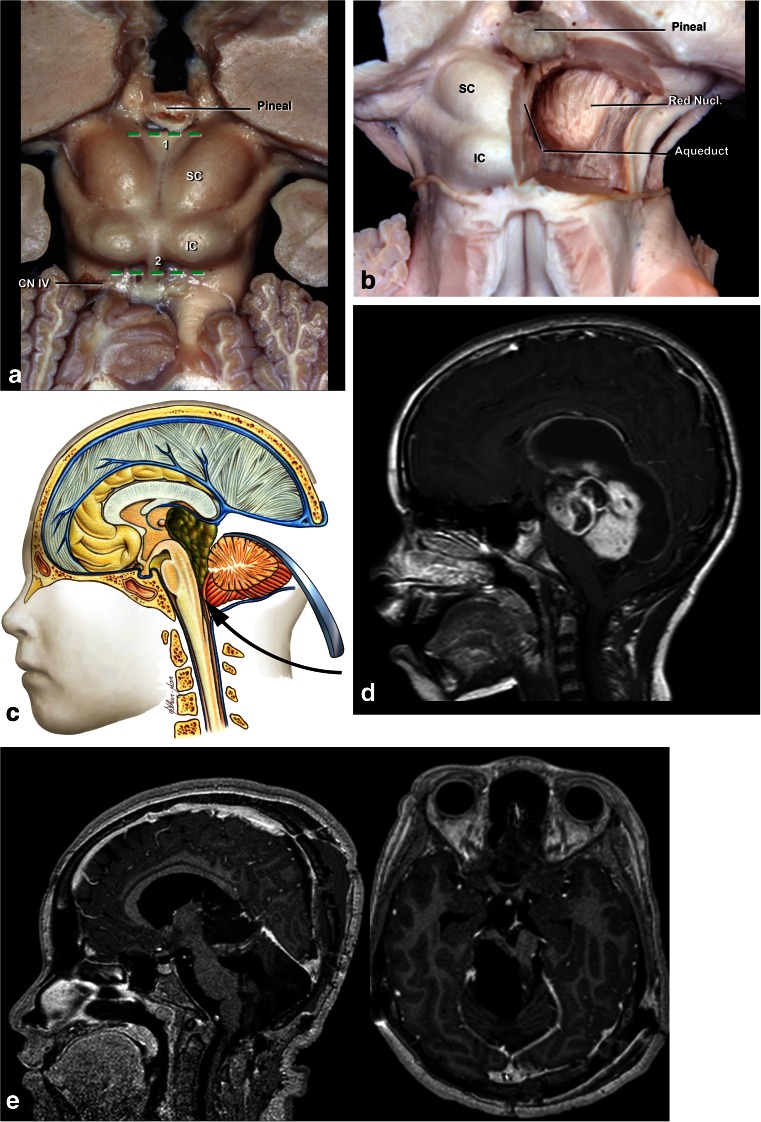
**a** Posterior (*dorsal*) midbrain is composed of pair superior (SC) and inferior colliculi (IC). The transverse supracollicular incision (*1*) is made just above the upper edge of the superior colliculus. The infracollicullar incision (*2*) is directed transversely between the CN IV and the lower edge of the inferior colliculus. **b** Further dissection of dorsal (posterior) midbrain. The important landmark is the cerebral aqueduct, which is located just behind the medial longitudinal fasciculus (MLF), oculomotor, and trochlear nuclei at the midline. The red nucleus extends from the midlevel of the inferior colliculus to the lateral wall of the third ventricle. **c** Cartoon of a large tumor growing towards third and fourth ventricle which can be approached by combined infratentorial supracerebellar followed by subocciptal telovelar approach. **d** A large pilocytic astrocytoma of the quadrigeminal plate in a 5-year-old patient, growing towards the third ventricle and fourth ventricle, approached via combined supracerebellar-infratentorial and telovelar pathway through the rhomboid fossa. **e** Postoperative MRI scan shows gross total resection

Some tumors may grow and require surgery. In such cases, two types of approach have been used: when they grow towards the third ventricle, the supracerebellar infratentorial route is chosen (Fig. 11); however, if they grow towards the superior part of the fourth ventricle, the transtentorial occipital approach proposed by Poppen [51] and modified by Ausman [3] (Fig. 10) has been preferred. The transtentorial occipital approach was first described by Horrax in 1937 [28], modified by Poppen in 1968 [51], and popularized by Jameson in 1971 [30]. Several surgical positions have been described, such as sitting, prone, concorde, and three-quarter prone. Ausmann (1988) [3] described the transtentorial occipital approach with a three-quarters prone body position, the side with the lesion turned down, the coronal plane at a 45-degree angle with the floor, and the head at 30-degree flexion and 15-degree elevation. This allows the occipital lobe to drop by force of gravity, and so there is less need of cerebral retraction, and the risk of homonymous hemianopsia as a consequence of retraction of the occipital lobe. This approach enables an excellent view of the pineal region, postero-lateral surface of mesencephalon, tentorial surface of the cerebellum, splenium of the corpus callosum, and posterior third of the third ventricle.

This approach is mainly recommended for tumors having a large superior and lateral extension, with the displaced venous complex impairing the view of the tumor through a posterior medial pathway.

In this approach, an occipital craniotomy is performed involving the occipital suture and bordering the transverse and superior sagittal sinus. The dura can be opened in a C-shape with the base facing the superior sagittal sinus, or by two triangles with the bases facing the superior sagittal and transverse sinuses. The cerebellar tentorium is opened parallel to the straight sinus for 1.5 to 2 cm. A small incision in the splenius of the corpus callosum may also be performed to enlarge the view of the tumors extending go the posterior third of the III ventricle. When the tumor grows towards the third ventricle and the superior part of the fourth ventricle, that region becomes a “blind” region—and therefore, we use the infratentorial supracerebellar, associated to the suboccipital telovelar approach in order to reach the fourth ventricle [4] (Fig. 11).

Therefore, for midbrain surgery, we have four “safe zones”: through the perioculomotor area for anterior region lesions, supracollicular access, infracollicular access, and through the lateral mesencephalic sulcus to the intermediate midbrain. Lesions of the posterior midbrain are usually exophytic, not requiring brainstem incision.

### Pons

Most pontine tumors are diffuse; therefore, resective surgery is not beneficial and chemotherapy/radiotherapy is indicated. Neurosurgeons must differentiate between focal, low-grade exophytic, and diffuse tumors for patients to benefit from surgery.

**Fig. 12:**
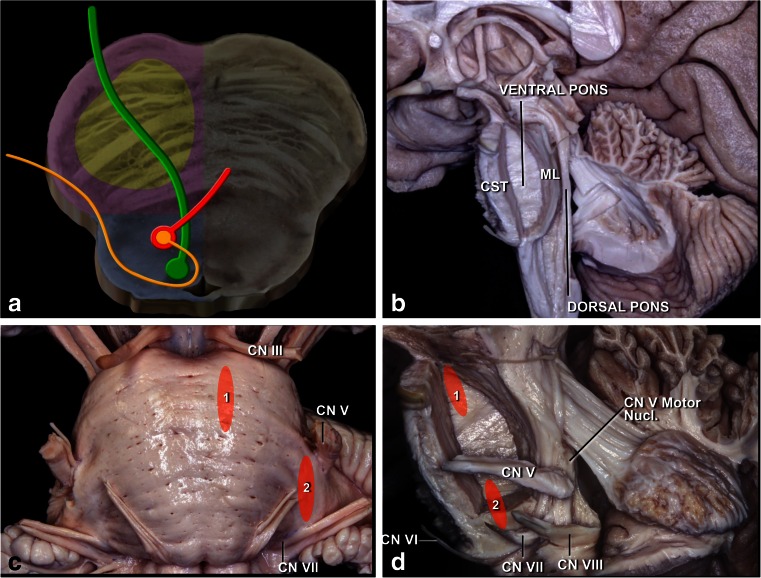
**a**. Topography of the corticospinal tract in the pons (*yellow*). Nuclei and courses of the VI (*green*) and VII (*orange*/ *red*) cranial pairs. **b** Lateral view. The ventral pons located in front of the medial lemniscus (ML) and the dorsal pons located behind the ML have been exposed. **c** Anterior view of the pons. The supratrigeminal (*1*) and peritrigeminal (*2*) safe entry zones are used for lesions located in the ventral pons. The supratrigeminal incision is made 4 mm below the pontomesencephalic sulcus at the same sagittal level as the exit point of the CN III. The peritrigeminal incision is made medial to the entry zone of the CN V and between CN V and CN VII. **d** Lateral view of the ventral pons. Neurocritical structures at risk are the corticospinal tract, trigeminal motor nucleus, and intrapontine segment of the CN V–VIII

The pons is located between the superior pontomesencephalic and inferior pontomedullary sulci. The pons is divided in two at the level of the medial lemniscus into anterior and posterior, or ventral and dorsal. The pons contains the pyramidal tract, which is more medial and anterior than in the midbrain, as well as the trigeminal, abducens, facial, and vestibulocochlear nerves and nuclei. Therefore, it is imperative to know the intrinsic and extrinsic anatomy of the fifth, sixth, and seventh cranial nerves in the pons (Fig. 12).

**Fig. 13:**
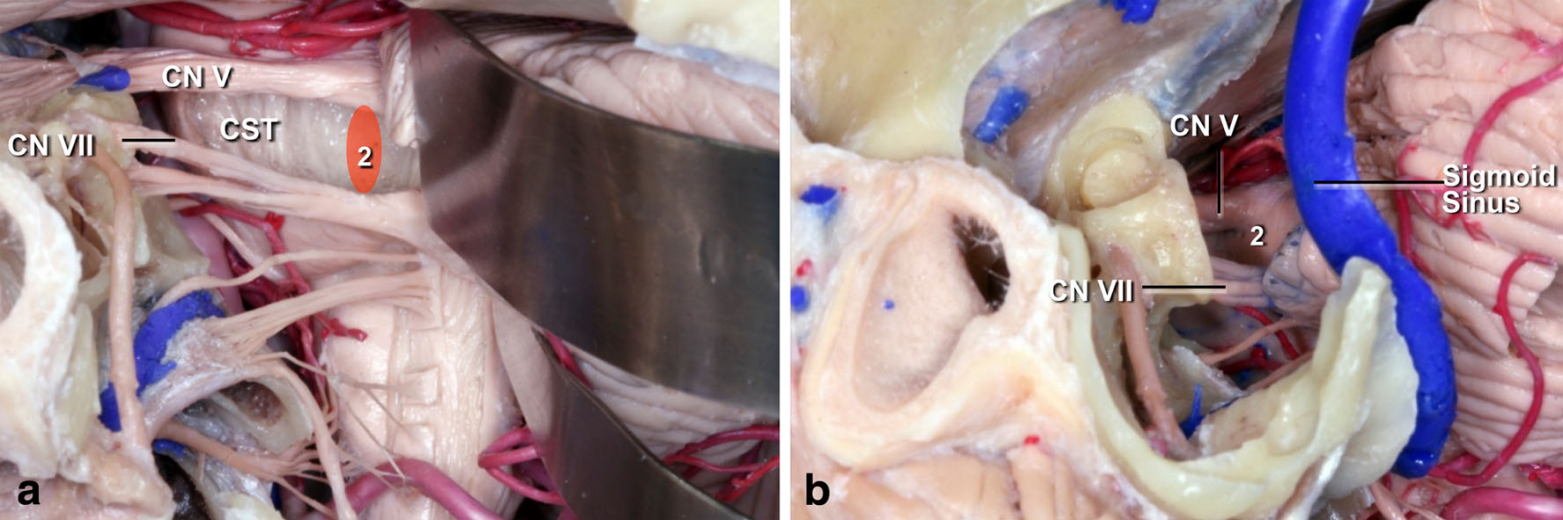
**a** The retrosigmoid approach can be used for supra- and peri-trigeminal entry zones. **b** The presigmoid approach provides good exposure for supra- and peritrigeminal entry zones

The facial nerve courses around the sixth nerve nucleus. This relationship must be very well established when tumors are approached via the rhomboid fossa. Three arterial groups provide blood to the pons: anteromedial, lateral, and dorsal. The anteromedial arteries are derived from the basilar artery and terminal vertebral artery branches. These arteries nourish the paramedian tegmentum (including the pyramidal tract fascicles), medial lemniscus, medial longitudinal fasciculi, reticular formation, and abducens nucleus. Perforating lateral branches arise from the superior cerebellar artery (SCA), anterior inferior cerebellar artery (AICA), and long pontine arteries. They supply the superior cerebellar peduncle, central tegmental tract, lemniscus side, locus ceruleus, motor and sensory main trigeminal nuclei, abducens nucleus, facial nucleus, superior olivary nucleus, pontine reticular nucleus, lemniscus side, and pyramidal tract. Terminal SCA branches comprise the posterior arterial supply of the pons. They perfuse the superior cerebellar peduncle, mesencephalic nucleus of the trigeminal nerve, and the locus ceruleus [55] (Fig. 13).

### Anterior pons

**Fig. 14:**
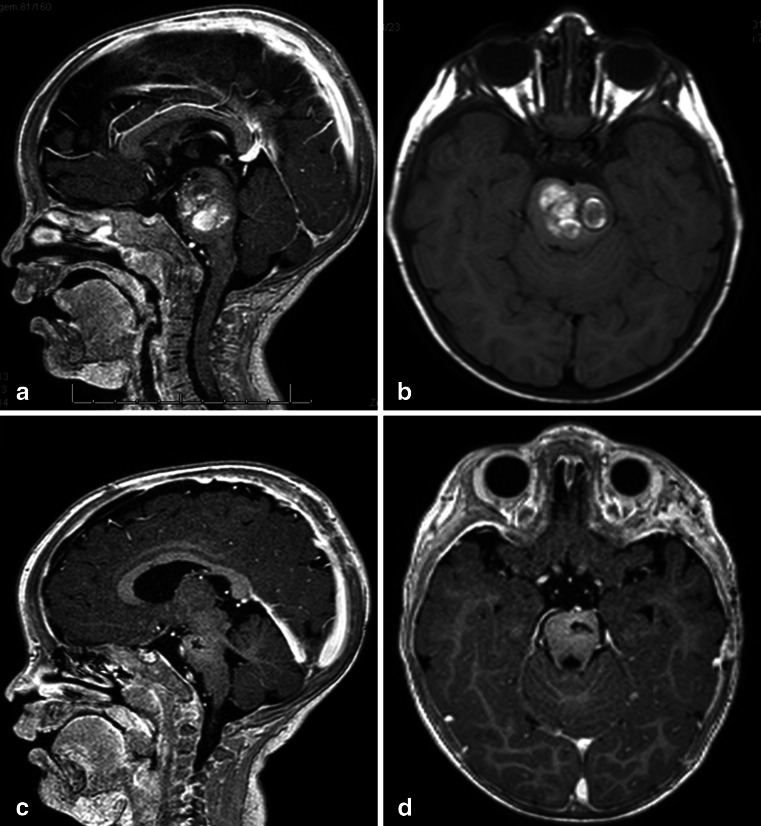
**a**, **b** 8-month-old patient with a large lesion in the anterior and superior portion of the pons, approached via orbito-fronto zygomatic via the supratrigeminal entry zone. **c**, **d** 6-month postoperative follow-up after complete resection of the lesion

Tumors of the anterior and upper pons can be accessed using an orbito-fronto-zygomatic route, which is a modification of the supraorbital craniotomy described by Jane et al. in 1982 [31]. The third cranial nerve is a key reference point in this approach. To expose the upper portion of the pons, it is necessary to dissect the interpeduncular and pre-pontine cisterns with durotomy of the free edge of the tentorium. The entry point is supratrigeminal. A 4-mm vertical incision is made below the mesencephalo-pontine sulcus in a line from the third to the fifth cranial nerve; thus, we named this access route “supratrigeminal.” We have used this route for superior ventral lesions without additional patient morbidity (Fig. 14).

**Fig. 15:**
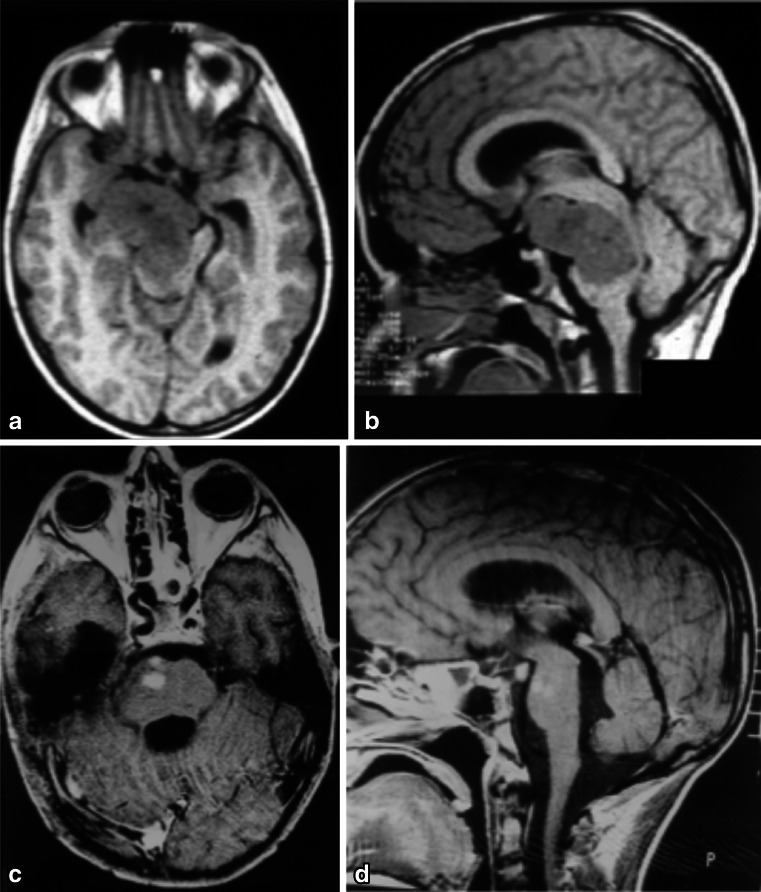
**a**, **b** 10-year-old patient with tetraparesis. Tumor operated with a pre-sigmoid approach. **c**, **d** Postoperative control 10-year after gross total resection, showing no evidence of tumor

For lesions located in the anterior and inferior portions of the pons, or for ventrolateral lesions, the pre-sigmoid approach has been used (Fig. 15). The incision in the pons is made longitudinally between the points of emergence of the fifth and seventh cranial nerves. However, this corridor is too narrow, good only for biopsies or for removal of cavernomas therein. This approach can be achieved through a paramedian occipital pathway or through the petrosal route. In the anterior approach of the pons, the region around the emergence of the fifth nerve is a safe area to be opened 1 cm wide at 1 cm from the midline, but one should be careful not to go very anterior to avoid the corticospinal tract.

### Posterior pons

**Fig. 16:**
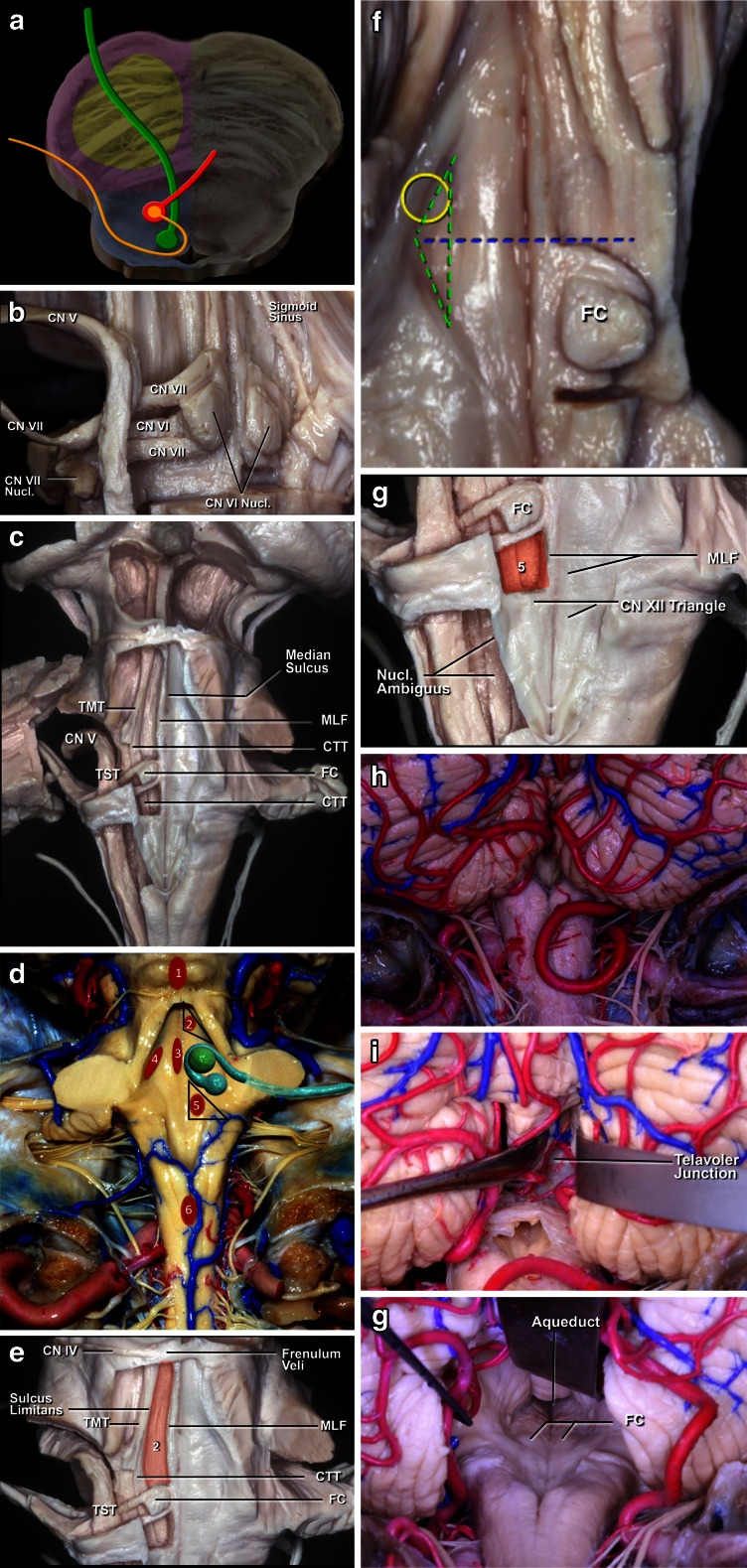
**a** Topography of the corticospinal tract in the pons (*yellow*). Nuclei and courses of the CN VI (*green*) and VII (*orange*/*red*) cranial pairs. **b** The facial colliculus, which is produced by the CN VI nucleus and intrapontine segment of CN VII, is the most important landmark on the floor of the fourth ventricle. **c** There are four important structures avoiding their damage: the medial longitudinal fasciculus (MLF), central tegmental tract (CTT), and trigeminal mesencephalic (TMT) and spinal (TST) tracts. **d** Posterior brain stem safe zones: *1* pericollicular, *2* suprafacial, *3* interfacial, *4* lateral sulcus limitans, *5* infrafacial, and *6* middle. **e** The suprafacial entry zone (*2*) is bordered superiorly by frenulum veli containing the CN IV, inferiorly by the facial colliculus (FC), medially by medial longitudinal fasciculus (MLF), and laterally by sulcus limitans, a lateral sulcus. **f** Superior fovea, is a depression formed by sulcus limitans, has a triangular shape (*green triangle*). The apex of this triangle is at the same axial level as the uppermost margin of the facial colliculus. The trigeminal motor nucleus (*yellow circle*) is located at a deeper point of the superolateral edge of this triangle. **g** The infrafacial entry zone is bordered superiorly by facial colliculus, inferiorly by hypoglossal (CN XII) triangle, medially by medial longitudinal fasciculus (MLF), and laterally by the CN VII nucleus and nucleus ambiguus. **h**. Median subcoccipital approach is used to reach the floor of the fourth ventricle. **i** Telovelar junction is incised. **j** Exposure of the floor of the fourth ventricle

Posterior pontine lesions are accessed via the rhomboid fossa (Fig. 16). Superior and posterior pontine lesions are accessed by a telovelar route, also known as a cerebellomedullary fissure approach [42, 43, 46]. The opening to the brainstem above the facial nerve is called the suprafacial triangle, which is defined medially by the medial longitudinal fasciculus (i.e., the median sulcus), caudally by the facial nerve (having the facial colliculus as reference), and laterally by the upper cerebellar peduncles. This triangle is approximately 1 cm^2^. Although it is a safe entry triangle, prudence recommends bipolar stimulation on the surface in order to localize the course of the facial nerve, which might have been deviated due to the growth of the tumor. Entry to this zone must always be 2 mm from midline to preserve the medial longitudinal fasciculus. However, recovery after injury of this fascicle is very fast compared to partial facial nerve involvement.

**Fig. 17:**
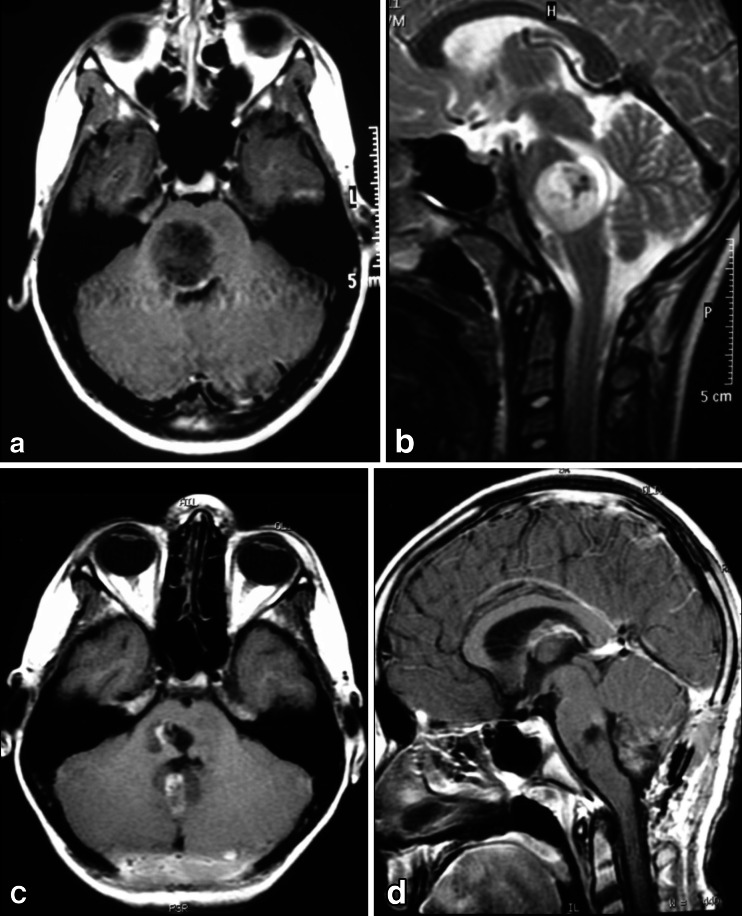
**a**, **b** Superior and posterior pontine tumor approached via suboccipital telovelar approach with the point of entry into the pons through the suprafacial triangle. Astrocytoma grade II. **c**, **d** Postoperative control showing gross total resection

This topography accepts a little retraction in the superior and lateral direction, while retraction in the caudal direction should be as far as possible avoided (Fig. 17).

**Fig. 18:**
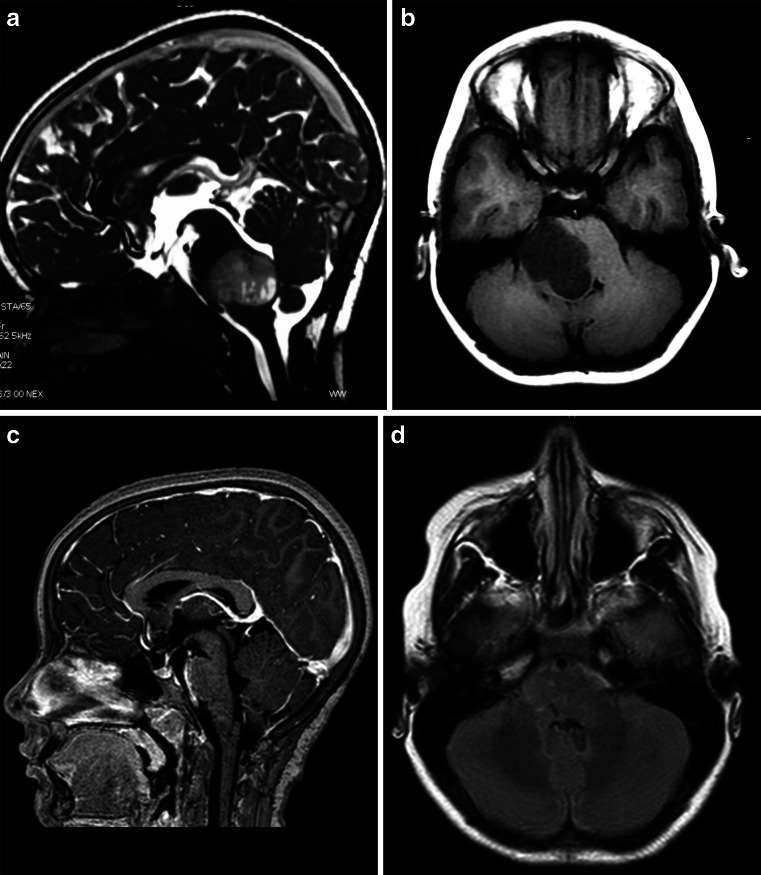
**a**, **b** 5-year-old patient with pontine lesion approached by infrafacial entry point. **c**, **d** 8-year follow-up. Pilocytic astrocytoma

For posterior and inferior lesions, the infracollicular route is used, through the infrafacial triangle (Fig. 18), which has as a medial border, the medial longitudinal fasciculus, and is bordered caudally by the medullary striae and laterally by the facial nerve.

This is a much smaller triangle and the safe distances are not always the same, so that intraoperative monitoring is mandatory. The safe area to access the infracollicular triangle as described by Kyoshima et al. [39] would begin on average 6.5 mm above the obex and would extend for 9.2 mm in the cranial-caudal direction; the supracollicular triangle would be on average 22.5 mm above the obex, with an extension of 13.6 mm.

We have used a third approach, when no space is found in the rhomboid fossa, namely the interfacial approach. Bricolo and Turazzi [7] have described the midline access in the rhomboid fossa as being possible at the level of the facial colliculi, next to the nucleus of the sixth nerve, since the fibers of the medial longitudinal fasciculi are not yet crossed at this level. In this approach, the medial longitudinal fasciculus is damaged, which may disturb the conjugate movement of the eyes. From the surgical point of view, we have used a bilateral telovelar approach, with coagulation of the choroid plexus of the fourth ventricle, which allows for ample access from the obex to the cerebral aqueduct without need to harm the cerebellar vermis.

More lateral lesions have been approached through the lateral sulcus limitans, also via a telovelar approach [40].

Lawton et al. [40] have proposed a supratonsillar approach to the inferior cerebellar peduncle, without the need to open the IV ventricle and perform an expanded telovelar approach. This route has been described for cavernomas, but it can be sufficient for tumoral lesions of the inferior cerebellar peduncle extending to the midline. This technique is best used with the aid of neuronavigation.

Therefore, for the pons, we have the following “safe zones”: supratrigeminal, peritrigeminal, suprafacial, infrafacial, interfacial, and through the lateral sulcus limitans.

### Medulla

**Fig. 19:**
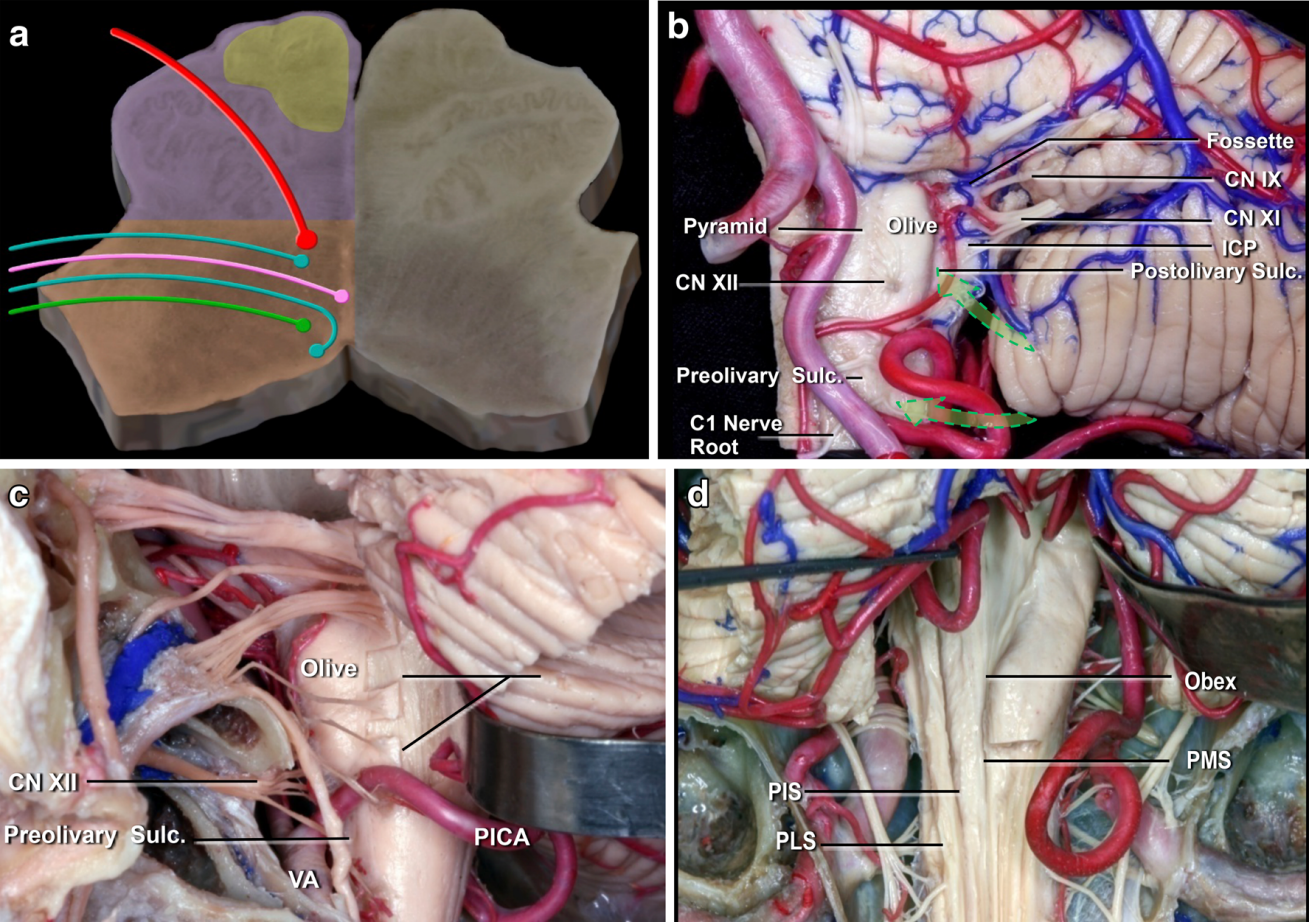
**a** Schematic representation of the medulla at the olive level, with the presence of the IX, X (*green and pink*), XI, and XII (*red*) cranial pairs nuclei and topography of the corticospinal tract (*yellow*), which is much nearer to the midline as compared to the pons and the midbrain. **b** Ventral medulla. The safe entry zones in the ventral medulla are the preolivary and postolivary sulci. The preolivary sulcus is located between the olive and the pyramid, which houses the CST. The depression rostral to the olive, the supraolivary fosette, is just below the junction of the facial and vestibulocochlear nerves with the brainstem. The glossopharyngeal, vagus, and accessory nerves exit the medulla just dorsal to the postolivary sulcus, which is located between the olive and inferior cerebellar peduncle (ICP). The hypoglossal rootlets exit the medulla along the preolivary sulcus. **c** The far lateral approach used for pre- and post-olivary sulci. **d** Dorsal medulla. The posterior median (PMS), intermediate (PIS), and posterolateral (PLS) sulci have been proposed as the safe entry zones. The suboccipital median approach

The medulla is the most caudal portion of the brain stem, and it is separated from the pons by the bulbopontine sulcus (Fig. 19). The inferior limit of the medulla is the pyramidal decussation and foramen magnum in the ventral surface. The posterior surface is the obex. It receives blood supply from the vertebral artery and branches of the anterior spinal artery. The anterolateral perforating arteries perfuse the pyramidal tract and the inferior olivary nuclei. The lateral arteries are branches of the posteoinferior cerebellar artery (P.I.C.A.), anteroinferior cerebellar artery (A.I.C.A.), and vertebral and basilar arteries, and they perfuse the inferior cerebellar peduncle, the spinothalamic tract, spinocerebellar tract, spinal trigeminal nucleus, central reticular formation, dorsal motor nucleus of the vagus, nucleus and tractus solitarius, and the hypoglossal, vestibular, cochlear, cuneate and ambiguous nuclei. The gracile and cuneate nuclei, area postrema, and vagal, solitary, and medial vestibular nucleus are supplied by these branches [55].

### Anterior medulla

**Fig. 20:**
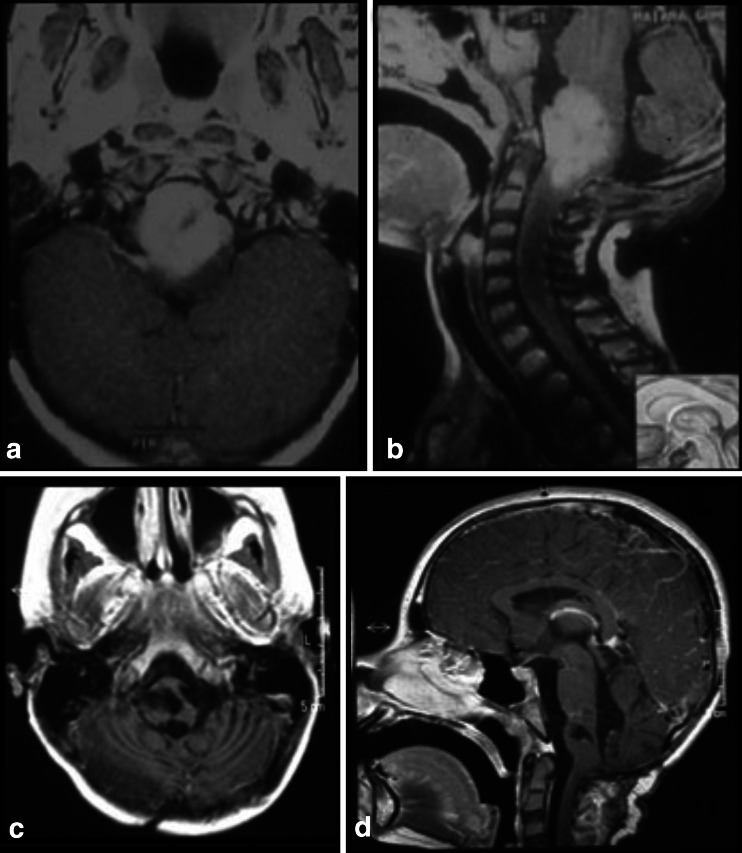
**a**, **b** 2-year-old patient evolving to tetraparesis. A large tumor anterior to the medulla is present. The far lateral approach and trans-olivary point of entry was used for resection of a pilocytic astrocytoma. **c**, **d** Eleven-year follow-up, showing no evidence of tumor

**Fig. 21:**
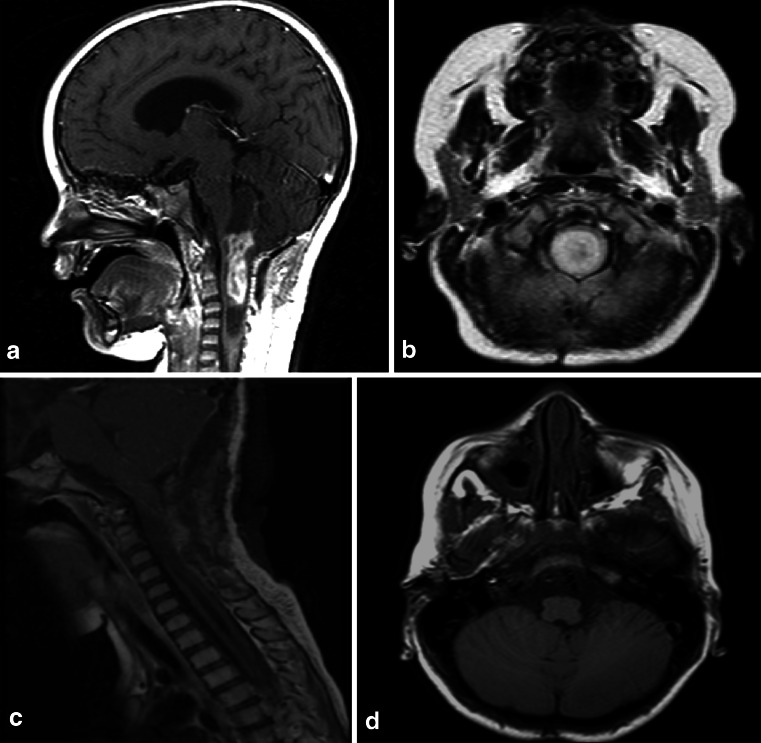
**a**, **b** Cervico-medullary tumor with exophytic extension in the fourth ventricle. **c**, **d** MRI at 5 years after surgery shows no evidence of tumor

The medulla is perhaps the most difficult structure to be approached, due to the high density of nuclei located therein, the cranial nerve pairs from IX to XII. Lesions located in the anterior portion of the medulla are accessed via a far-lateral approach. This approach was initially described by Heros [27] and by George et al. [21] among approaches to the craniovertebral junction. There are many variations of approaches according to the part of condyle to be removed: transcondylar, supracondylar, and paracondylar exposure [56]. In children, it is possible to access the anterior portion of the medulla without removing the condyles (Fig. 20). The section of the dentate ligament next to the entry of the vertebral artery facilitates mobility of the medulla, and so the lateral access becomes easier, as it avoids opening the condyle. Access to the brainstem may be anterior to the olive, posterior to the olive, or sometimes through the olivary body, preferably in the postero-olivary sulcus (Figs. 20 and 21). It is possible to enter the medulla via the anterolateral sulcus. This entry zone is along the pre-olivary sulcus, between the caudal hypoglossal and the rostral C1 rootlets. It lies very near the pyramidal tract, next to its decussation, and should be used only for exophytic lesions [9]. The retro-olivary sulcus is a safe entry area. According to Recalde et al. [53], the olivary body offers a surgical space of approximately 13.5 mm in the craniocaudal axis, 7 mm in the transverse axis, and 2.5 mm in the anterodorsal axis. The entry zone is through the post-olivary sulcus located between the olive and the inferior cerebellar peduncle ventral to the glossopharyngeal and vagus rootlets [53].

### Posterior medulla

Intrinsic lesions in the posterior part of the medulla are difficult to approach due to the huge quantity of nuclei in that region. On the other hand, most of lesions therein have an exophytic component, which facilitates the access. These tumors are called cervico-medullary. Medullary lesions inferior to the obex may be accessed via the midline through the posterior median sulcus, as are the intramedullary lesions.

In the intraoperative period, severe vegetative alterations may occur, such as hypertension and tachycardia in the case of medullary lesions on the right side and bradycardia for medullary lesions on the left side.

## Results

From 1991 to 2011, we evaluated 303 patients less than 18 years old with tumors located in the brainstem treated in the Pediatric Neurosurgery Service and Institute of Pediatric Oncology of São Paulo Federal University. Ninety-six cases were diffuse tumors that underwent chemotherapy/radiotherapy. The first author of this article operated on the remaining 207 patients, which corresponds to 9.1 % of all patients operated by this service in the same period (2015 patients). Ages ranged from 8 months to 18 years, with a mean age of 10 years. All “safe zones” previously described were used in these patients, and a new “safe zone,” termed supratrigeminal, was used in three patients for tumors of the anterior and superior pons. One hundred patients were operated without intraoperative monitoring, while 107 cases were monitored. The 207 operated cases are summarized in the Table 1.

**Table 1:** Distribution of the 207 operated cases according to topography, approach, safe zone entry point, and morbidity/mortality

Topography	Division	Approach		Safe zone entry point	Morbidity/mortality
Midbrain (84)	Anterior (16)	Transventricular transforaminal endoscopic approach (6)		Exophytic lesion	None
		Pterional or fronto-obito zygomatic transylvian approach (10)		Peri-oculomotor (10)	None
	Median (59)	Infratentorial supracerebellar (53)	Median (41)	Supracollicular and/or infracollicular	-Air embolism (2) -Hypertensive Pneumoventricles (10) -Rubral tremor (2)
			Paramedian (12)	Lateral mesencephalic sulcus	
		Infratentorial supracerebellar combined with telovelar approach (6)		Supracollicular and/or infracollicular	None
	Posterior (9)	Occipto-transtentorial (8)		Exophytic lesion	None
		Infratentorial supracerebellar combined with telovelar approach (1)			
Pons (72)	Anterior (11)	Fronto-obito zygomatic transylvian approach (3)		Supratrigeminal (3)	None
		Presigmoid (8)		Peritrigeminal (8)	None
	Posterior (61)	Suboccipital craniotomy with telovelar approach (61)		Suprafacial (45)	-MLFI (6) -Facial paresis (3) -Death (1)
				Interfacial (3)	-MLFI* (2)
				Infrafacial (13)	Facial paresis (9)
Bulb (51)	Anterior (11)	Far-Lateral Approach (11)		Transolivar	None
	Posterior (40)	Suboccipital craniotomy with Telovelar approach (40)		Midline	-Death (3) -Breathing and swallowing impairment (9) -Vocal cord incoordination (2)

*MLFI* medial longitudinal fasciculus injury

Eighty-four patients had midbrain tumors. Sixteen were located in the anterior portion of the midbrain, 59 in the central midbrain, and nine in the quadrigeminal plate. Six patients with tumors located in the anterior portion extending to the third ventricle were operated on by pure neuroendoscopy, coupled with an ultrasonic aspirator device (Sonoca 300 and 92–030 micro handpiece—Söring). All of the lesions were exophytic, and there was no need to incise the brainstem. The tumors did not bleed much and could be easily aspirated with the ultrasonic aspirator at low power. The main clinical manifestation in these patients was intracranial hypertension due to hydrocephalus. All tumors were pilocytic astrocytomas, and removal was completed with no case requiring a ventricular shunt. Ten patients with tumors in the anterior portion of the midbrain extending to the interpeduncular fossa were operated via the transsylvian approach. Six accesses were achieved via classic pterional for exophytic tumors and four via fronto-orbito-zygomatic, and access to the brainstem was lateral to the third nerve (perioculomotor access). Regarding the central midbrain (59 cases), 53 patients were approached using a supracerebellar infratentorial entry in the sitting position, with 41 cases median and 12 paramedian. In six patients, access was achieved through a combined median supracerebellar infratentorial route with sub-occipital telovelar access through the rhomboid fossa. For the medial supracerebellar infratentorial approaches, the pre-central cerebellar vein was coagulated in all the cases, and no complication ensued. In the paramedian access, coagulation of the pre-central cerebellar vein was not necessary, and the entry point to the brainstem was through the lateral mesencephalic sulcus. Two patients had air embolisms, and aspiration through a central venous catheter was needed to resolve the issue. The air entry point was in the transverse sinus adjacent to the sigmoid sinus, which is the highest and most lateral point of opening in the dura mater. Ten patients had hypertensive pneumoventricles, of which two underwent neuroendoscopy for treatment. Two patients had rubral tremors controlled with clonazepam. Nine patients had tumors in the quadrigeminal lamina, eight of whom were operated via three-quarters prone, and one via a combined infratentorial supracerebellar and sub-occipital telovelar approach. All tumors of the midbrain were low-grade astrocytomas, most of them pilocytic astrocytomas.

Of 168 patients presenting with pontine tumors, 96 were diffuse and not operated upon. Seventy-two tumors in this topography were operated. Four cases had tumors located in the anterior and superior pons, seven in the anterior and inferior pons, 48 in the superior and posterior pons, and thirteen in the inferior and posterior portion

Three tumors located in the anterior and superior pons were operated on using a fronto-orbital-zygomatic route with the brainstem entry point being supratrigeminal, between the third cranial nerve and trigeminal nerve, 4 mm below to the mesencephalon-pontine sulcus. One case was operated on using the pre-sigmoid approach. None of these patients with tumors in the anterior and superior portion of the pons had morbidity. In seven cases, the tumors were situated in the anterior and inferior portion of the pons and were accessed via a presigmoid route. Forty-eight tumors were located in the posterior and superior pons, while thirteen were located in the posterior and inferior pons. All tumors located in the dorsal part of the pons were operated on using a telovelar route through the rhomboid fossa with patients in the prone position. Forty-five tumors in the superior and posterior pons were approached by a suprafacial pathway, and three cases using an interfacial pathway above the facial colliculus. In six patients operated using a suprafacial route, there was injury of the medial longitudinal fasciculus and facial paresis in three cases. In all cases, the symptoms regressed 6 months after surgery. One patient developed acute hydrocephalus and died 3 days later. In the three cases operated on using the interfacial pathway, two developed symptoms of medial longitudinal fasciculus injury that disappeared within 6 months. No facial nerve involvement was observed. Thirteen patients had tumors in the posterior and inferior portion of the pons and were operated on via the infrafacial route. Eight of these patients had facial paralysis, with five cases resulting in permanent paralysis. Twenty-two cases were grade III and grade IV astrocytomas, while 50 cases were low-grade astrocytomas.

Fifty-one patients had medullary tumors, with 11 located in the anterior and 40 at the so-called cervicomedullary junction. Anterior tumors were accessed using a far-lateral approach with an entry point through the olivary medullary route, or where the exophytic tumor resided. Dentate ligament resection was performed in all cases using this route. Cervicomedullary junction tumors were operated on using a telovelar route with the medulla incised longitudinally in the midline. Nine patients showed worsened breathing and swallowing. Three patients remained in respiratory failure and died of pneumonia within 2 months of surgery. Two patients required permanent tracheostomy due to vocal cord incoordination. Of the medullary tumors, eight were gangliogliomas, three were hemangioblastomas, 29 were astrocytomas, eight were grade III and IV astrocytomas, and 21 were low-grade astrocytomas.

For the entire series of 207 surgically treated tumors of the brainstem, operative mortality seen in the first 2 months after surgery was 1.9 % (four patients) and surgical morbidity occurred in 21.2 % (45 cases). All tumors of the midbrain in our series were low-grade astrocytomas. In the pons, 50 had low-grade astrocytomas, and 22 were high-grade tumors. Of 51 medullary tumors, only eight were high-grade astrocytomas. Thus, of 207 children with tumors of the brainstem, 30 (14.4 %) were high-grade tumors, while 177 were of low grade of malignancy. The follow-up ranged from 3 to 20 years with a mean of 13 years. The disease-free survival (5 years) for benign tumors was 92 %, while median survival for high-grade gliomas was 18 months. Table 1 summarizes the patients and the safe zone entrance used in the 207 patients.

## Discussion

Tumors of the brainstem are more common in children than in adults. Few publications address surgical approaches to brainstem tumors in children. Most of them are related to brainstem approaches for surgical treatment of cavernomas. Surgery for removal of a cavernoma in this location is far different from surgery to remove a brainstem tumor. A peculiar feature of cavernomas is that the cavity produced by widening Virchow-Robin spaces and the consolidation of clots causes a large space after the removal of the clot, facilitating cavernoma resection. Therefore, small incisions at the surface of the brainstem are sufficient to remove bulky cavernomas. In pediatric tumors of the brainstem, the first disadvantage is working on a very small structure compared to the brainstem of an adult, except with rare cystic lesions. On the other hand, many tumors are exophytic, allowing for complete removal without incising the brainstem.

Improvements in diagnosis, with high resolution MRI combined with tractography, are very helpful for surgical decisions as well as for the choice of the safest and more precise surgical approach. Advances of neuroanesthesiology, intraoperative electrophysiological monitoring, and intensive postoperative care, give more security, allowing for more aggressive surgical excisions and preventing damage [61].

Neurosurgical instruments have also evolved significantly. Today, we have microscopes with high brightness and such definition as to allow improved recognition of where the tumor ends and normal tissue begins. Lighter and more delicate instruments with diamond tip scalpels allow precise incisions of the brainstem. The routine use of ultrasonic surgical aspirators with 1-mm tips allows us to remove large lesions through small openings in the brainstem. Surgical planning of the most appropriate means of access, associated with intrinsic and extrinsic knowledge of brainstem anatomy, is also key to achieving success in surgery.

The brainstem is routinely divided into three parts: midbrain, pons, and medulla [41]. We further divided the brainstem into seven parts: anterior, central, and posterior midbrain; anterior and posterior pons; and anterior and posterior medulla, which helped us to choose the best surgical approaches [11, 12].

Cantore et al. [9] divided the brain stem into two surgical planes—the anterior and the posterior. Thus, they classify six regions in the brain stem. Our reason for dividing the mesencephalon into three portions is because the quadrigeminal plate tumors characteristics are different from those of the anterior and central midbrain. Most tumors in the quadrigeminal plate are indolent and rarely have to be operated upon, restricting treatment to hydrocephalus control [13].

Four “safe entry zones” for the midbrain have been described. The most complex and anterior, called the perioculomotor zone, has been described by Bricolo et al. [8] There is a space between the oculomotor nerve and the pyramidal tract which can be accessed through an incision parallel and lateral to the oculomotor nerve. The presence of a tumor may increase this distance, facilitating surgery. When tumors have an exophytic component, the brainstem can be directly entered through the tumor. Clinically, these patients present preoperatively with third cranial nerve paralysis and contralateral pyramidal involvement (Weber syndrome) that usually disappears quickly after lesion removal. Small tumors only cause diplopia.

In our series, 16 tumors were located in the anterior portion of the mesencephalon. Six were growing towards the III ventricle and were operated on by pure neuroendoscopy, coupled with an ultrasonic aspirator device. We found few literature reports of brainstem tumor resection using purely neuroendoscopic methods. Miki et al. have used the neuroendoscopic trans-third ventricle approach in six cases for lesions of the ventral brainstem surface [45], but just in one case for brainstem tumor.

In ten patients of our series, tumors grew towards the interpeduncular cistern. Six of these tumors were exophytic, large, and were approached by a classic pterional route with opening of the Sylvian fissure and lesion removal. Wide opening of the Sylvian fissure in young children is sometimes difficult, and simple delicate manipulation can cause vasospasm, thus papaverine is often used. In four patients, the tumors were intrinsic, and the brainstem was approached through the perioculomotor route. In all the cases, a fronto-orbito-zygomatic approach was used. We may also use the temporopolar access described by Sano in 1980 [58], which provides an anterolateral view of the interpeduncular fossa. These are the access routes we use for the anterior portion of the midbrain, entering through the third ventricle, or through the perioculomotor space. Konovalov and Kadyrov [35] proposed a transchoroidal temporal access to these anterolateral lesions located in the midbrain, especially on the dominant side and when tumors extend to the ambient cistern. The fact is these lesions are quite rare in this topography. In our series, only 7 % of the tumors considered surgical were located in the anterior portion of the midbrain. Although Albright 1993 [2] reported that only 7 to 8 % of brainstem tumors are located in the midbrain, in Yasargil’s [64] series of 167 brainstem tumors, 26 (15.5 %) were located in the midbrain, double that reported by Albright [2]. Garzon et al. [20] found that 33.8 % of brainstem tumors were located in the midbrain. In our series of 207 cases considered surgical, 84 were located in the midbrain (40.5 %), but if we consider all cases of brainstem tumors treated by our service, this rate drops to 27.7 %. We believe this is because we are a neurosurgical center of reference, not being forwarded cases of diffuse pontine tumors.

Of 59 patients with central midbrain tumors operated through an infratentorial supracerebellar routes described by Krause in 1911 [36], 41 had a median and 12 a paramedian route. When approaching via the paramedian route, we used the same access as the median approach; the only difference being that the brainstem is accessed via the lateral mesencephalic sulcus. Six patients had tumors growing not only towards the third ventricle but also towards the fourth ventricle, and they were operated on, via a combined approach, namely an infratentorial-supracerebellar followed by a suboccipital telovelar approach across the fourth ventricle. All patients were operated on in the sitting position with the head bent. Wide craniotomy of the posterior fossa was performed and the dura opened to avoid the occipital sinus, which is usually patent in young children. The C1 arch was removed in all cases. Yasargil [64] advocates a different access to this region by not removing the arch of C1 and opening the dura mater 2 cm below and parallel to the transverse sinus, thus preventing further exposure of the cerebellum to avoid possible herniation. In our series, we had no cerebellar herniation. The vermian veins, which are bridging veins between the tentorial face of the cerebellum and the tentorial and transverse sinus, were coagulated without any complication [55]. When necessary, we also coagulated the precentral cerebellar vein without any clinical repercussions. In two cases, the patients had a symptomatic air embolism which was resolved with blood aspiration though the central line and identification of the venous opening in the apex of the dura mater incision close to the transverse sinus.

Nine patients with quadrigeminal plate tumors underwent surgery, eight via the occipital transtentorial route and one through combined infratentorial supracerebellar and suboccipital telovelar routes. In four cases, entries were supracollicular, and five were infracollicular, above the fourth cranial nerve. For tumors of the central or posterior portion of the midbrain growing in the direction to the third ventricle, we used the supracerebellar infratentorial approach. For tumors growing into the fourth ventricle, we use a transtentorial occipital access in the three-quarter position. When the lesions grew into the third and fourth ventricles, we used the combined access route.

Ogata and Yonekawa [47] proposed a paramedian infratentorial supracerebellar access route for lesions of the superior, intermediate, and lower cerebellar peduncle. They demonstrated that it is possible to open the surface of the intermediate cerebellar peduncle lateral to the cerebellar mesencephalic fissure.

The approaches to the anterior portion of the pons are the most difficult. Bagahai et al. [5] have described a safe entry zone to the ventrolateral portion of the pons between the output points of the fifth and seventh cranial nerves. This corridor, however, is very narrow and is good only for biopsy or removal of cavernomas in that region. This access can be reached via an occipital paramedian approach or through a petrous access. In other access routes to the anterior pons, the region around the emergence of the fifth cranial nerve is a “safe” area that may be opened 1 cm wide and 1 cm from the midline. However, one should be careful not to go further anterior to avoid the corticospinal tract.

Eleven of our patients had tumors in the anterior pons. Four were anterior and superior, and seven were anterior and inferior. Three anterior and superior tumors were accessed via a fronto-orbito-zygomatic approach, and the point of entry into the lesion was a vertical incision, 4 mm inferior to the mesencephalopontine sulcus in the same direction as the third nerve. In three patients operated using this route, there was no increased morbidity. This approach does not have a similar description in the literature, and we prefer to call it “supratrigeminal access” to differentiate it from peritrigeminal access between the fifth and seventh cranial nerves. In fact, this new entry point is between the third and fifth cranial nerves medial to the pyramidal tract. Other tumors of the ventral pons were approached using a pre-sigmoid route, with ligature of the superior petrosal sinus.

Sixty-one tumors were located in the posterior pons, 48 of them being superior and 13 inferior. The superior tumors were approached either via the suprafacial triangle as proposed by Kyoshima et al. [39], or, in lateral cases, through the lateral sulcus limitans. In three patients, because of distortion caused by the tumor in the rhomboid fossa, it was extremely difficult to locate an entry point by stimulating the facial nerve. Therefore, the entry point was an interfacial access superior to the facial colliculus, as described by Bricollo and Taruzzi [7]. However, the medial longitudinal fasciculus was injured in two patients. Thirteen patients with inferior pons tumors were operated on through the infrafacial triangle. Eight patients operated by this route developed facial paralysis, which was permanent in five of them. We believe that this route should be reserved only for small exophytic tumors or cavernomas. Access through the rhomboid fossa, below the medullary striae, should also be avoided as this region has a high density of cranial nerve nuclei. All access to the rhomboid fossa was performed using a suboccipital route with the removal of the C1 arch and telovelar approach, sometimes bilaterally, to avoid opening the cerebellar vermis. The patient’s position was ventral decubitus.

Medullary tumors present the same technical difficulty as the tumors of the pons. The anterior ones were approached via suboccipital far lateral craniotomy with removal of the C1 arch and section of the dentate ligamentum which is close to the vertebral artery entry into the skull. That section of the ligament has permitted further mobility of the medulla, facilitating identification of the olivary body and entry into the brain stem through the posterior olivary sulcus (or transolivary entry). Right-sided medullary access usually produced intraoperative hypertension and tachycardia, whereas access from the left side produced bradycardia. These natural alarms must not be suppressed with drugs, such as atropine or hypotensive drugs, because they reveal that we are manipulating the brainstem too aggressively. These are indirect physiological alarm that requires attention. Usually, these vegetative storms occur when the lesion is under traction, which should be avoided. Instead, we should remove the tumor using an ultrasonic aspirator, avoiding traction. These small vegetative storms are not reasons to interrupt procedures, because they cease immediately upon stopping the traction and irrigation with warm saline.

Lesions situated in the posterior portion of the medulla were also operated via occipital telovelar approach, the medulla being reached opening the midline below the obex, through the posterior medial sulcus.

There are eleven previously described “safe” entry zone areas in the brainstem. In this article, we describe another zone, the “supratrigeminal,” for anterior and superior pons lesions. This route was used in three patients and showed no morbidity. It is therefore a reasonable access route. However, a fronto-orbito-zygomatic access is needed for wide visualization of the mesencephalopontine sulcus and the third cranial nerve.

It is up to neurosurgeons to choose correctly pathways for complete lesion resection with a minimum morbidity. However, morbidity is still high (22 % in our series). Hydrocephalus is always a catastrophic complication in the postoperative period of brainstem tumors, and the neurosurgeon should always be aware of this possibility. One of our patients died of hydrocephalus. Thus, whenever possible, we place an external ventricular drain that is removed in the first 48 h postoperatively.

The brainstem does not permit either traction or coagulation; thus, we routinely use ultrasonic aspirators with little aspiration and delicate tips, only performing bipolar coagulation as a last resort with abundant irrigation since the heat may damage the brainstem delicate structures.

## Conclusions

**Fig. 22:**
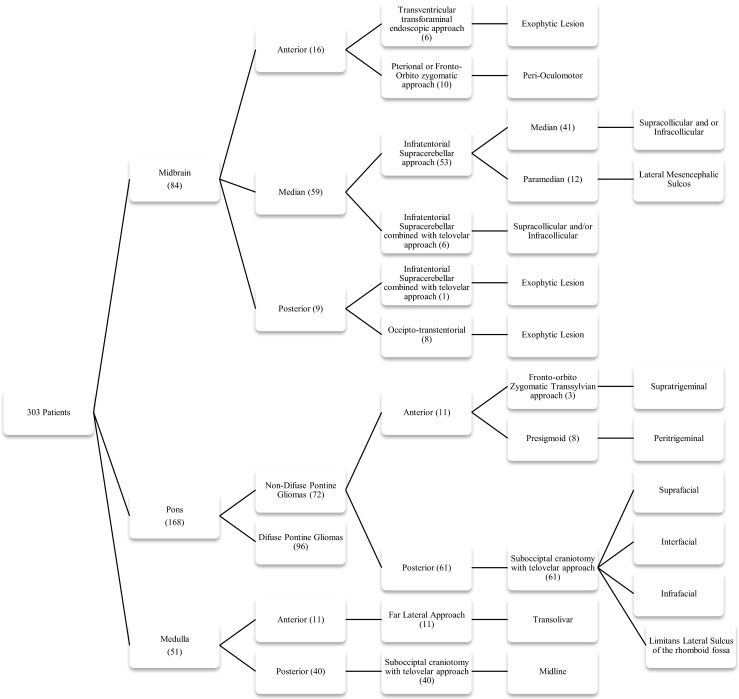
The algorithm to approach the safe zones

Brainstem surgeries are among the most difficult in neurosurgery, especially for pediatric neurosurgeons, as brainstem tumors are more frequent in childhood than in adult life. Although imaging with tractography has evolved significantly, they still do not show the nuclei and nerve pathways inside the brainstem. Anatomic dissection by the Klingler’s technique to study white fibers has helped neurosurgeons in planning the three-dimensional architectural design of surgical approaches [1, 34]. There are eleven previously described “safe entry zones” in the brainstem: perioculomotor, the lateral mesencephalic sulcus, infracollicular access, supracollicular access, peritrigeminal, suprafacial, interfacial, infrafacial, lateral sulcus limitans, periolivary, and the posterior median sulcus. A new safe entry zone has been used in three of our patients, the “supratrigeminal” zone, for approaching anterior and superior pontine lesions. Fig. 22 summarizes the algorithm to approach the safe zones. Maybe in the near future, with the improvement of high- resolution 7 Tesla MRI, or another type of imaging able to show the precise deviations caused in the brain stem structures by effect of the tumors, coupled with a more efficient electrophysiological monitoring system, this kind of surgery may become simpler with less morbidity. Currently, more accurate knowledge about intrinsic/extrinsic anatomy of the brainstem associated with more sophisticated tools, like high-resolution microscopes and ultrasonic aspirators with thinner tips, have allowed us to remove a large number of brainstem tumors with acceptable mortality and morbidity rates from any topography in this wonderful cerebral structure.
